# Effect of Lumbar Spinal Stabilization Exercises Along With Neural Tissue Mobilization on Pain and Spinal Dysfunction in Failed Back Surgery Syndrome

**DOI:** 10.7759/cureus.72396

**Published:** 2024-10-25

**Authors:** Pradnya Dhake, Sandeep Shinde, Sawani Aphale

**Affiliations:** 1 Department of Musculoskeletal Sciences, Krishna College of Physiotherapy, Krishna Vishwa Vidyapeeth Deemed to be University, Karad, IND

**Keywords:** back surgery, lumbar-fusion, manual muscle strength, obesity, spinal stabilization

## Abstract

Background

Failed back surgery syndrome (FBSS) mainly involves back pain radiating to the lower limb after back-related surgeries. It can develop various complications around the operation site and its surrounding area. This study evaluates the effect of lumbar spinal stabilization exercises and neural tissue mobilization on pain and spinal dysfunction in FBSS.

Objectives

To study the impact of lumbar spinal stabilization exercises and neural tissue mobilization on pain and spinal dysfunction in FBSS. To compare the impact of lumbar spinal stabilization exercises and neural tissue mobilization with conventional therapy on pain and spinal dysfunction in FBSS.

Methods

The study included 76 participants aged 45-60 with back pain radiating to lower limbs after back-related surgeries within the past six months. Participants were randomly assigned to either a conventional treatment group (Group A) or an experimental exercise program group (Group B). The treatment duration was six weeks and data were analyzed by a paired t-test for within-group analysis and an unpaired t-test for between-group analysis.

Results

Both groups A and B showed significant differences in all three outcome measures. However, Group B showed extremely significant improvement (<0.0001) in outcome measures, including pain, lumbar muscle strength by manual muscle testing, and modified Oswestry index assessment compared to Group A.

Conclusion

Both conventional and experimental groups showed a significant impact on the pain and level of function in failed back surgery syndrome patients. However, lumbar stabilization exercises along with neural tissue mobilization (Group B) showed a more pronounced impact on all outcomes such as pain reduction and improvement in lumbar flexor, rotator, and extensor muscle strength and modified Oswestry index compared to the conventional treatment group (Group A).

## Introduction

Back pain is highly prevalent in the population, which influences multiple factors such as psychological, social, and financial aspects in life. It becomes more prevalent with an increase in age. Therefore, in some cases, to treat the root causes, it is understandable that the number of back surgeries is increasing. As previous research stated, in annual surgery counts, lumbar fusion surgeries are the most frequently performed [1]. Patients who had surgery related to back complications have experienced chronic back pain, with or without radicular symptoms. These patients had one or more surgeries to treat the pain in the past [2,3]. As per the previous study records, around 20-40% of the patients had persistent pain post-spinal surgery [4].

Many factors can cause failed back surgery syndrome (FBSS). Among the preoperative factors, obesity is considered an important factor. As we know, lumbar vertebrae take more load during activity than other vertebrae. In the case of obese patients, it will not be possible as obesity will restrict movement during the activity. This will lead to pain at the operation site [5]. Patients who are going to have surgery may experience altered emotional status before surgery. Patients may face depression, anxiety which in turn leads to poor recovery postoperatively. As per the previous study, Patients should be encouraged to deal positively with the hospitalization which will help to progress the patient's condition faster. Coping strategies should taught to patients in these conditions helping to recover [6].

In the postoperative cases, the surgery related to the back pain can cause various biomechanical changes in the region, resulting in increased load over the adjacent structures. This can cause changes primarily degenerative in the area of spinal fusion above and below [5]. Altered biomechanics can strain the lower back muscles, putting tension on them while controlling the movement of the spine. This ultimately results in stiffness, muscle spasms, inflammation at the operation site, fatigue, and increased pain in the paraspinal area [7].

Intraoperative factors also affect the condition. A single-level decompressive surgery performed on a patient with pain occurring due to multiple-level spinal involvements is unlikely to relieve the pain [8]. Intraoperative errors can cause new trigger point sources that can cause pain [9]. Due to all these factors, pain and restriction in performing daily activities will convert into long-term complications [9,10]. 

FBSS can cause back pain and dysfunction of the spine along with pain radiating to lower limbs or in some cases it may not be radiating. The pain will lead to various compensatory patterns during movements. Treating and alleviating the pain will lessen postural complications [11]. Many postural changes like a flat back, increased thoracic kyphosis, inability to stand steadily for a prolonged time, and compensatory movement patterns during lumbar spine movements occur due to incision. There may be spasms and atrophy in the patient that will affect posture. It can cause hyperextension of the thoracolumbar spine which causes poor posture and long-term pain. Postoperatively various changes can occur like segmental lordosis, general lordosis, and sacral slope [12,13].

Evidence is present and they focused on treatments according to symptoms it suggests that electrotherapy modalities with exercises for strengthening are useful to relieve the pain and strengthen the muscles. This particular treatment works on the symptoms and not the cause. For complete relief of pain, the root cause which is dysfunction in major cases needs to be treated [11,14].

Neural tissue mobilization is passive and applied by the therapist to decrease mechanical stress. Mobilization of peripheral nerves will improve the efficiency of nerve conduction [15]. Exercise for pain in the lower back helps to maintain spinal stability [15]. Core stabilization is the main and important factor in the exercise regime. Core muscles like multifidus, erector spinae, quadratus lumborum as well and paraspinal muscles are targeted during exercises aimed at improving neuromuscular control, endurance, and strength of muscles [16]. Postoperatively, scar formation is a natural part of the healing process. Spine surgery results in the formation of adhesions at the operative site (In the area surrounding the lower lumbar vertebrae). These adhesions can cause back and lower limb pain by compressing the nerve roots and reducing the range of motion due to a lack of movement [17]. Postoperatively, patients reduce their activities as they believe that exercise will cause discomfort and increase pain. Limited physical activity results in a loss of muscle strength and flexibility. Stabilization exercises decrease the pain and improve stabilization in the lumbar area [18,19].

Therefore, the purpose of this study is to evaluate the effect of lumbar stabilization exercises along with neural tissue mobilization on pain and dysfunction in FBSS. This study aims to determine whether these therapeutic interventions can provide significant relief from symptoms and facilitate better outcomes in patients with FBSS.

## Materials and methods

**Figure 1:**
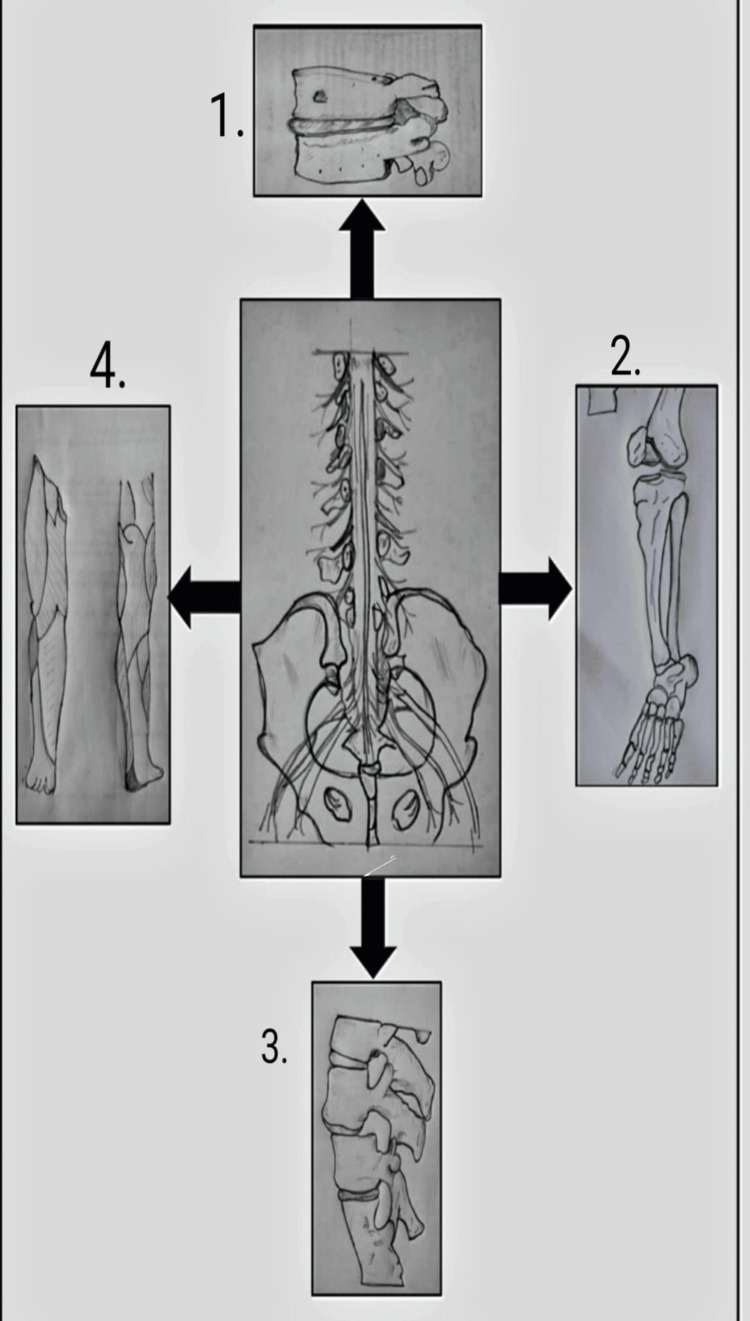
Diagrammatic representation of complications that occur due to failed back surgery syndrome 1. Nerve root compression; 2. Affected L5 nerve root; 3. Disc herniation; 4. Lumbar radiculopathy The concept of these diagrams are taken from following sources: centre diagram: [21], diagram 1, 4: [22], diagram 2: [23], diagram 3: [24]

As per Figure 1, various post-failed back surgery complications are explained in a diagrammatic representation. Complications that occur are as follows: 1. Nerve root compression - When a nerve is trapped or compressed by surrounding tissues, it can cause numbness or tingling sensation in the affected part; 2. Affected L5 nerve root - The L5 nerve root supplies to the group of muscles in the pelvis and legs including tibialis anterior, extensor hallucius longus, extensor digitorum longus, and the lateral head of the gastrocnemius. An affected nerve root can cause complications like foot drop [20]; 3. Disc herniation - This occurs when the outer layer of the disc in the spine bulges out. Malalignment of vertebrae may occur in some cases; 4. Lumbar radiculopathy- it may be due to a herniated disc or bone spur formation that presses on the nerve and can cause radiating lower limb pain.

This study was conducted to evaluate the impact of neural tissue mobilization with lumbar stabilization exercises on FBSS patients. The study's purpose and procedures were thoroughly explained to all participants, and written informed consent was obtained. A preliminary assessment was conducted before the main evaluation. This comparative study was carried out at Krishna College of Physiotherapy, Karad. The study was approved by the Institutional Ethical Committee (protocol no. 107/2022-2023). A total of 76 participants were enrolled, with 68 completing the treatment through to the conclusion of the study. Eight patients were dropped out of the study for various reasons. Participants were divided using a simple random sampling procedure. They were divided into each group by using the envelope method. Two envelopes containing the group names (A and B) were made and participants were asked to pick an envelope just before the commencement of the intervention. The intervention allocated to participants in the envelope was given to the participant. Thirty-four participants in Group A received conventional treatment protocol. Thirty-four participants in Group B received lumbar spinal stabilization along with neural tissue mobilization for pain and spinal dysfunction in FBSS. The sample size was calculated by: n = Z^2^ pq/L^2^; Where n = sample size, Z = standard normal variant at 95% (1.96), p = proportion of obese individuals, q = 100-p, L = allowable error 

The study participants were both male and female patients aged 45-60 years with a history of lumbar fusion surgery of more than six months to two years, having pain in the lower back radiating to the lower limb post-surgery. Criteria for exclusion were patients with a history of tumor or infection of the spine, joint disorders, cardiopulmonary disorders, and diagnosed psychiatric disorders. The data was collected using the Visual Analogue Scale (VAS), Manual Muscle Testing, and the Modified Oswestry Disability Index Questionnaire as outcomes. The assessment was taken before and after the study, and the scores were noted for each patient. 

Outcomes

VAS is a measurement instrument that tries to measure a characteristic or attitude believed to range across a continuum of values and cannot easily be directly measured. The VAS is a single-dimension tool for assessing pain intensity and is widely used in various adult populations [25]. The Oswestry Low Back Disability Questionnaire is a highly regarded instrument utilized by researchers and disability evaluators to evaluate the extent of permanent functional disability in patients. Widely acknowledged as the "gold standard" for assessing functional outcomes in cases of low back pain, it collects essential information regarding the impact of back pain on an individual's capacity to perform daily activities [26]. In manual muscle testing there is a significant grading system of muscle testing. According to the Oxford grading system will measure the muscle. In this system, grading is from zero to five. Zero indicates no movement, one is flickering of movement, two is a movement with gravity assistance, three is a movement against gravity, four indicates movement against gravity with minimal resistance, and five signifies movement against gravity with full or maximum resistance [27]. In manual muscle testing for lumbar extensors, the patient should be in a prone position with his hand clasped behind head. The therapist's hand stabilized the lower extremity at the ankle, and another hand was used to stabilize the pelvis. Instruct the patient to perform extension until the entire thorax is raised above the table. A point at which the patient has sufficiently cleared the umbilicus from the table is optimum muscle strength. For lumbar flexors, the patient should be in a supine position with hands clasped behind head. The patient flexes the trunk until scapulae clear the table. A point at which the patient has sufficiently cleared the scapulae off the table is optimum muscle strength. For lumbar rotators, the patient should be in a supine position with hands clasped behind head. The patient flexes trunk and rotates to one side, the same will be performed for the opposite side. The scapula of the opposite side of rotation should clear the table which is a point of optimum muscle strength.

The treatment protocol was given to groups A and B.

Conventional Group

Treatment was given three times a week for two weeks and afterward, the patient was instructed to perform the exercises at home three times a week for three months following the exercise regime with progression. A hot pack and interferential therapy (IFT) were provided for 20 minutes. In Table 1, various exercises were included such as pelvic bridging, curl-ups, straight leg raises, resistance band exercises in supine like resisted hamstring curls, and leg presses with thera band were used for lower back and lower limb strengthening. Also, heel slides, hamstrings, and quadriceps isometrics were given. The exercises were progressed according to repetitions for up to six weeks.

**Table 1:** Treatment protocol given to group A

WEEKS	EXERCISES	REPETITIONS
WEEK 1-2	Pelvic bridging exercises	5
	Curl up exercises	5
	Straight leg raise	5
	Resistance band exercises (In supine )	5
	Heel slides	5
	Hamstrings and quadriceps isometrics (5 second hold)	5
WEEK 3-4	Pelvic bridging exercises (Single leg)	10
	Curl up exercises	10
	Straight leg raise ( with half kg )	10
	Resistance band exercises (In supine )	10
	Heel slides	10
	Hamstrings and quadriceps isometrics (5 second hold)	10
WEEK 5-6	Pelvic bridging exercises (Single leg) – 5 second hold	10
	Curl up exercises	10
	Straight leg raise (with one kg)	10
	Resistance band exercises ( In supine)	10
	Heel slides	10
	Hamstrings and quadriceps isometrics – 10 second hold	10

Experimental Group

The selected subjects were given spinal stabilization exercises along with neural tissue mobilization. Exercises were repeated three times a week for six weeks. Before starting the exercises, a hot pack and IFT were given to the patient for 20 minutes. These exercises followed a specific principle which was breathing in and out, carefully and gradually pulling in the lower abdomen below the umbilicus, while keeping the upper stomach, back, and pelvis still, which leads to what is known as hollowing [28]. In Table 2, the following exercises were explained. In abdominal bracing, the subject is in a crook lying position. Keep the inward curve in your lower back. Breathe normally throughout. The legs were placed in a neutral position, with the feet shoulder-width apart, the hip joint at 45°, and the knee joint at 90°. It involves contracting the abdominal muscles as though preparing to be struck in the stomach. While this causes the abdomen to naturally draw in slightly, the exercise itself does not require actively pulling the stomach in or pushing it out. Abdominal bracing exercises (ABE) engage all the layers of abdominal muscles, including the deep muscles in the lower back, such as the transversus abdominis and multifidus, contributing to core stability [29]. In back isometrics exercises, the subject is in the supine position. Place a roll of towel below the lumbar region and hold it for five seconds. After a count of five seconds, repeat the same activity [30]. In arm leg raise exercise, subjects maintain the quadruped position, then raise one arm, return to the quadruped position, and then raise one leg. For progression, raise both the arm and leg simultaneously [31]. For ball squat, place the ball between the wall and your lower back, walking your feet out slowly. Lower your body toward the squat position while continuously pushing back against the ball. Straighten your back by keeping your weight on your heels while standing [28]. For side-lying hip lift, the subject's position is in supine lying. The other leg should be in the knee-flexed position. Ask the patient to lift the hip in an upward direction. Hold for five seconds and then relax [32].

**Table 2:** Treatment protocol given to group B

WEEKS	EXERCISES	REPETITIONS
WEEK 1-2	Abdominal bracing	5
	Back isometric exercise	5
	Arm leg raise with alternately arm and limb raising	5
WEEK 3-4	Abdominal bracing	10
	Back isometric exercise	10
	Arm leg raise with alternately arm and limb raising	10
	Side lying hip lift	5
WEEK 5-6	Abdominal bracing	10
	Back isometric exercise	10
	Arm leg raise with alternately arm and limb raising	10
	Ball squat	10
	Side lying hip lift	10

For neural tissue mobilization for the tibial nerve, the therapist stood next to the plinth, facing the patient’s feet. The patient’s feet were positioned in dorsiflexion and eversion, maintained while applying overpressure during knee extension, and any symptoms were recorded. With dorsiflexion, eversion, and knee extension held, the therapist passively raised the leg into hip flexion until reaching the point of initial tension. To further engage the proximal sciatic nerve, hip adduction, and internal rotation were introduced. In this position, neural tissue mobilizations using the straight leg raise technique were performed, involving nerve flossing in a controlled, graded manner. The mobilizations were performed slowly and rhythmically for two sets of 10 repetitions per session. As the patient’s condition improved, attempts were made to increase the range of motion and joint positioning [33]. For the peroneal nerve, the therapist stood next to the plinth, facing the patient's feet. The patient's foot was placed in plantar flexion and inversion, which was maintained while applying overpressure during knee extension, with symptoms being observed. While holding plantar flexion, inversion, and knee extension, the therapist passively raised the leg into hip flexion until initial tension was felt. To further increase traction (sensitization) on the proximal sciatic nerve, hip adduction, and internal rotation were added to the straight leg raise. In this position, neural tissue mobilizations involving nerve flossing were performed, with adjustments based on the condition's severity and irritability. The mobilizations were carried out in a controlled on/off pattern, with two sets of 10 slow and rhythmic repetitions in each session [33]. 

The test assessment was conducted using the VAS, Oswestry Back Disability Questionnaire, and Manual Muscle Testing. Patients who completed their treatment for FBSS were given this interventional protocol. Follow-up was done for six weeks. The interpretation of this research study was completed based on comparing pre-test and post-test assessments of all the outcome measures.

Statistical analysis

Statistical analysis of the obtained data was completed with the help of IBM SPSS Statistics software (SPSS version 25; IBM, Armonk, NY, USA). A paired t-test was used to compare outcome parameters before and after intervention. The arithmetic mean and standard deviation were calculated for each outcome measure. The arithmetic mean was obtained by adding all the values and dividing by the total number of entries and the software was also employed to calculate the respective percentages.

## Results

Table 3 demonstrates all the demographic data consisting of age, gender, working status, habits, and obesity correlated with the patients. The study includes 34 patients aged 45-60 years in each group. The study includes both 20 males (29.41%) in each group and 14 females (20.58%). It consists of both the working (26.47%) and the non-working population (23.52%) in each group. According to body mass index (BMI), patients were categorized as overweight (30.88%) in Group A, (29.41%) in Group B, and obese category included (19.11%) in Group A and (20.58%) in Group B

**Table 3:** Demographic Data BMI- Body Mass Index

Demographic variables	Category	Number of patients (Group A)	Percentage (Group A)	Number of patients (Group B )	Percentage (Group B)
Age	45-60 years	34	50.00%	34	50.00%
Gender	Male	20	29.41%	20	29.41%
	Female	14	20.58%	14	20.58%
Working status	Workers	18	26.47%	18	26.47%
	Non workers	16	23.52%	16	23.52%
BMI	Overweight	21	30.88%	20	29.41%
	Obese	13	19.11%	14	20.58%

Table 4 provides the before and after values of VAS for groups A and B. For Group A, the VAS (at rest) post-p value was <0.0001, and the VAS (on activity) post-p value was <0.0001. The mean difference for Group A pain (at rest) was 0.483 and for Group B, it was 1.35. For Group B, VAS (at rest) and VAS (on activity) post p value was <0.0001 which is considered extremely significant. The mean difference for pain (on activity) was 3.123 (Group A) and 5.53 (Group B). It was stated that the mean difference for pain reduction was greater in Group B than in Group A. t value for pain assessment is also mentioned in the table.

**Table 4:** Pain assessment by visual analogue scale VAS - Visual Analogue Scale; p-value - probability value

VAS ( at rest )	Pre test	Post test	Mean difference	P value	t value
Group A	2.494 ±0.909	2.011±0.815	0.483	<0.0001	2.31
Group B	2.317± 0.970	0.967±0.851	1.35	<0.0001	8.13
VAS (on activity)	Pre test	Post-test	Mean difference	p-value	t value
Group A	7.564±1.139	4.441±0.9595	3.123	<0.0001	17.33
Group B	7.238 ±1.267	1.708 ± 0.669	5.53	<0.0001	31.96

Table 5 states the muscle strength of lumbar flexors, extensors, and rotators on the left and right sides. The mean difference for Group A for flexors was 0.353 (p-value <0.0061), and for Group B it was 0.941 (p-value <0.0001). The mean difference for extensors, for Group A was 0.324 (p-value <0.0130), and for Group B, it was 0.852 (p-value <0.0001). The mean difference for rotators (right side) for Group A, was 0.147 (p-value 0.2296), and for Group B, it was 0.882 (p-value <0.0001). The mean difference for rotators (left side) for Group A, was 0.293 (p-value 0.0208), for Group B, it was 0.853 (p-value <0.0001). t value for manual muscle testing is also mentioned in the table.

**Table 5:** Manual muscle strength testing of lumbar muscles p-value - probability value

MMT(Lumbar Spine)	Pre -test	Post – test	Mean difference	p-value	t value
Flexors : Group A	2.470± 0.0506	2.823 ± 0.520	0.353	0.0061	5.57
Group B	2.5 ± 1.159	3.441 ± 0.560	0.941	<0.0001	6.03
Extensors: Group A	2.470 ± 0.506	2.794 ± 0.538	0.324	0.0130	3.62
Group B	2.647 ± 0.54	3.499 ± 0.564	0.852	<0.0001	9.00
Rotators:(Right side) Group A	2.470 ±0.507	2.617 ± 0.493	0.147	0.2296	1.71
Group B	2.617 ± 0.493	3.499 ± 0.507	0.882	<0.0001	10.31
Rotators:(Left side ) Group A	2.412 ± 0.499	2.705 ±0.523	0.293	0.0208	3.35
Group B	2.617 ± 0.493	3.470 ± 0.506	0.853	<0.0001	9.95

Table 6 demonstrates the Modified Oswestry Disability Index assessment. It interpreted the pre and post-results of groups A and B. The mean difference for Group A was 17.23 (p-value <0.0001), for Group B, it was 40.59 (p-value <0.0001). The mean difference between Group B was more than that of Group A. t value for the modified Oswestry Disability Index is also mentioned in the table.

**Table 6:** Modified Oswestry Disability Index Questionnaire p value - probability value

Modified Oswestry Disability Index	Pre-test	Post-test	Mean difference	P -value	t value
Group A	65.14 ± 2.298	47.91 ±2.667	17.23	<0.0001	40.49
Group B	66.23 ± 3.542	25.64 ±3.892	40.59	<0.0001	63.84

Table 7 demonstrates the between-group analysis (pre-test values) for the three outcome measures taken. P value for pain (at rest) 0.4403, pain (on activity) 0.2671. For lumbar flexor strength, it was 0.7324, and for extensor strength, it was 0.1678. For rotator strength (right side and left side), the values were 0.2296 and 0.0208 respectively. For the Modified Oswestry Disability Index, the value was 0.1395. The table also demonstrates between group analysis (post-test values) for the three outcome measures taken. The p-value for pain (at rest) and (on activity) was <0.0001. For lumbar flexor strength, the p-value was <0.0001. For rotators, the p-value was <0.0001; for lumbar extensors, it was <0.0001; for the modified Oswestry disability index, the p-value was <0.0001. As per the analysis, a significant difference was observed between the post-assessment values of the two groups. t value for both within and between group analysis is mentioned in the table.

**Table 7:** Within and between group analysis for all three outcome measures p-value - probability value

Pre-test values	Group A	Group B	P value	t value
Pain ( at rest )	2.494 ±0.909	2.317± 0.970	0.4403	0.77
Pain (On activity)	7.564±1.139	7.238 ±1.267	0.2671	1.11
Lumbar strength Flexors	2.470 ± 0.0506	2.513 ± 1.159	0.7324	0.21
Extensors	2.470 ± 0.506	2.647 ± 0.54	0.1678	1.39
Right rotators	2.470 ±0.507	2.617 ± 0.493	0.2296	1.21
Left rotators	2.412 ± 0.499	2.705 ±0.523	0.0208	2.36
Modified Oswestry Disability Index	65.147 ± 2.298	66.23 ± 3.542	0.1395	1.50
Post-test values	Group A	Group B	P value	t value
Pain ( at rest )	2.011±0.815	0.967±0.851	<0.0001	5.16
Pain (On activity)	4.441±0.9595	1.708 ± 0.669	<0.0001	13.62
Lumbar strength Flexors	2.823 ± 0.520	3.441 ± 0.560	<0.0001	4.71
Extensors	2.794 ± 0.538	3.499 ± 0.564	<0.0001	5.27
Right rotators	2.617 ± 0.493	3.499 ± 0.507	<0.0001	7.27
Left rotators	2.705 ±0.523	3.470 ± 0.506	<0.0001	6.12
Modified Oswestry Disability Index	47.911 ±2.667	25.64 ±3.892	<0.0001	27.52

## Discussion

FBSS is a chronic characterized by symptoms such as pain and radiating pain that persist for more than six months after surgery. This condition significantly impacts the patient’s quality of life through pain and dysfunction [2,19]. The study included 68 participants from Krishna Hospital, Karad, who were screened based on the inclusion criteria. All participants were informed about the study, and prior consent was obtained. A six-week follow-up was conducted. Group A received conventional physiotherapy, while Group B underwent an experimental physiotherapy treatment. The outcome measures included the VAS, Manual Muscle Testing, and the Modified Oswestry Low Back Disability Questionnaire. The results indicated that Group B (the experimental group) demonstrated a more significant improvement compared to Group A in patients with FBSS.

Previously Ahmed et al. conducted a study about 42 patients suffering from sciatica, assessed using a numerical pain rating scale (NPRS) and a 12-item short-form health survey (SF-12 questionnaire). The treatment was given to two groups, group A received sciatic nerve mobilization. Group B was given conventional physiotherapy. Sciatic nerve mobilization showed a significant impact in sciatica patients improving the pain and functional status of patients [33]. Evidence supports that the use of lumbar spinal stabilization exercises in populations with chronic back pain is effective. It stated that lumbar stabilization exercises improve pain and function in patients with chronic low back pain. These stabilization exercises also help strengthen core muscles [34].

Also, in another study conducted by Karahan et al., the subjects were divided into four groups, the first group was treated with isokinetic exercises, the second group with dynamic and static stabilization exercises, the third group with home exercises program, and the fourth group was the control group. They concluded that advanced exercise programs, including isokinetic and dynamic lumbar stabilization exercises, were more effective in enhancing physical function and psychological well-being. They further noted that dynamic stabilization exercises were superior to both isokinetic exercises and home-based exercises. Dynamic lumbar stabilization exercises specifically improved the range of motion in flexion and extension. Therefore, they concluded that stabilization exercises are a more effective approach for addressing back pain and dysfunction in patients with FBSS [35].

As per the previously conducted study, it was concluded that lumbar dynamic stabilization exercises were effective after lumbar-related surgery. Forty-two patients with disc herniations were operated on using the microdiscectomy method and were considered as samples. There were three groups involved. The first group was given dynamic stabilization, the second group was given home exercises, and the third group was the control group. Dynamic lumbar stabilization exercises helped to relieve pain, improving all functional parameters and strengthening of trunk and abdominal muscles [36].

One study conducted previously stated that slider, as well as tensioner neural mobilization exercises given along with transcutaneous electrical nerve stimulation (TENS), were more effective for back pain radiating to the legs than the control group. The study included 51 participants and the outcomes used were VAS, range of motion for the straight leg raise test, and the slump test for assessment. Neural tissue mobilization is used to regain the motion of neural tissue, restore homeostasis, and improve function [37]. 

Stabilization exercises are a more effective treatment option to improve back pain and lumbar dysfunction, enhancing both pain relief and functional status as well as muscle strength. In this study, patients were taught to perform lumbar spinal stabilization exercises in a safe, independent, and pain-free manner which was beneficial to improving pain, muscle strength, and quality of life. Neural tissue mobilization is more effective than conventional physiotherapy alone and also helping to improve pain, minimizing functional disability. Lumbar stabilization exercises aim to improve neuromuscular control, strength, and endurance of the muscles that are essential for maintaining dynamic spinal and trunk stability.

Strengths of the study

The study gives a holistic approach by combining neural tissue mobilization with lumbar stabilization which offers comprehensive rehabilitation addressing both nerves and muscle-related complications. By performing these exercises, the patient will show an active role in recovery which will improve the patient's self-efficacy. The study had some limitations: It was carried out in a specific area and the sample size was small. Suggestions and recommendations for future studies include examining different types of lumbar surgeries other than fusion surgery. This study can done in various geographic areas, involving larger populations and more outcomes. Studies with longer duration of treatment and long-term follow-up can be recommended in the future.

## Conclusions

This study concluded that lumbar spinal stabilization exercises and neural tissue mobilization have significantly improved in FBSS patients compared to conventional treatment. Also, it has a clinically beneficial impact on relieving pain and improving muscle performance. It is effective in reducing dysfunction and improving the function of the FBSS population. The study also stated that both the groups showed improvement in patient’s condition in pain and level of function outcomes but the experimental group showed a more significant impact than the conventional group in all three outcomes.
